# A robust conversion method of radioactivities between plastic and NaI scintillation well counters for long-term quality control and quality assurance

**DOI:** 10.1186/s40658-016-0154-3

**Published:** 2016-08-15

**Authors:** Takeshi Hara, Tadahiko Ishihara, Takahiko Asano, Masayuki Matsuo, Tetsuro Katafuchi, Hiroshi Fujita

**Affiliations:** 1 Department of Intelligent Image Information, Graduate School of Medicine, Gifu University, 1-1 Yanagido, Gifu, 501-1194 Japan; 2 Department of Radiology, Gifu University School of Medicine, 1-1 Yanagido, Gifu, 501-1194 Japan; 3 School of Health Science, Gifu University of Medical Science, 795-1 Ichihiraga, Nagamine, Seki, Gifu 501-3892 Japan

**Keywords:** Well counter, Scintillator, QAQC, Cerebral blood flow, SPECT

## Abstract

**Purpose:**

The purpose of this study is to specify a simple procedure for a robust data conversion of radioactivity value between plastic scintillator (PL) and NaI scintillator (NaI) devices.

**Materials and methods:**

The radioactivity estimate of 100 blood samples was measured by the two devices. The two radioactivities were plotted on the same graph. The least-squares method was applied to obtain the conversion function. The differences between the actual radioradioy (*N*) from the NaI device and the estimated radioactivity for NaI (*N*’) from the PL device activity (*P*) were statistically analyzed.

**Results:**

*N*’ was determined from *P* as *N*’ = 4.45 *P* + 6.28 with high correlation (*r* = 0.997). The Bland-Altman analysis between *N*’ and *N* showed no fixed bias and no proportional bias.

**Conclusions:**

A hundred blood samples using a fixed type of sample tubes and a fixed radionuclide may be required to set up the robust conversion function.

## Introduction

Quality control and quality assurance (QA/QC) processes are required not only for imaging cameras but also for peripheral devices [1]. Assessment of the radioactivity concentration in blood samples obtained in conjunction with brain scintigrams gives various quantitative parameters for analyzing the images from single photon emission computed tomography (SPECT). The results from the analysis are employed for diagnosis of various diseases related to blood flow in brain such as dementia or moyamoya diseases. The diagnosis, prognosis, and treatment often require long periods such as 5 to 10 years. The quantitative analysis for the patients’ images should be performed with high accuracy. In initial and repeat examinations, the radioradioy in the blood samples is sometimes used as an input value for a quantitative analysis. An example is an analysis using the quantitative SPECT (QSPECT) dual-table autoradiographic (DTARG) method [2]. The measurement of the radioactivity is performed by using a well counter. The sample radioactivity is estimated based on counting the photons emanating from the scintillation phenomenon in the crystal detector, but the number of counts often varies depending on the type of crystal detector and the counting software. Periodical calibrations of the devices maintain quantitative accuracy for the measurements for a while; however, replacement of the device will happen eventually in both laboratories and hospitals. After any change in the type of well counter, the difference in the measurement result will affect the quantitative analysis of images when the examinations were performed before and after the change. Using a cross calibration is generally the way to compare the results between two devices, and Nakajima et al. have reported on a method using phantom materials from 84 institutions to compare the heart-to-mediastinum ratio from ^123^I-metaiodobenzylguanidine scintigram images to realize a multicenter comparison [3, 4]. Yoneda et al. have reported the reproducibility of CBF estimations between two institutions employing different gamma cameras but have not mentioned the well counters employed [5]. Thus, cross calibration for well counters has not been reported on yet, since the measurement results may be consistent ones because of system calibration. The purpose of this study is to specify the number of samples and a simple procedure for a robust data conversion of radioactivity value between two devices and to elucidate the number of samples required.

## Materials and methods

### Devices and the input-output characteristics

We employed two well counters. One had a plastic scintillator (DCM-200, Hitachi-Aloka medical, PL) and the other a NaI scintillator (ARC-300, Hitachi-Aloka medical, NaI). Both of the devices were calibrated in April 2012. After the calibration process, the fundamental linearity of radioactivity estimate at the device output was confirmed independently for both by using 25 samples of various dilutions of ^123^I-IMP in water.

### Blood samples and measuring counts

We employed blood samples using the same sample tubes collected at one-point arterial blood sampling for the brain QSPECT DTARG method. The data collection was performed from April 5, 2012 to March 26, 2013 with an institutional review board (IRB) approval in Gifu University. The requirement for obtaining informed consent of individuals in this study was waived by the IRB because radioactivity in blood samples cannot be used to identify personal information. Data collection from the patient’s database was also approved by the IRB (#25-171). During the collection period, 100 of 107 DTARG examinations were performed correctly, and the 100 samples were used in this study. Seven samples were excluded because taking the blood samples failed in DTARG examinations. The mean age was 62.5 years; the ratio of male to female was 64:36. The counts from each sample were obtained by using the two devices sequentially with decay correction based on the time between the injection of the patient and the particular measurement. All the data for DTARG examinations were collected retrospectively because the samples in the periods were assessed by using the two different devices to prevent data loss and to avoid device flaws. With the NaI device, the energy window for the measurement was 159 ± 20 (keV). With the PL device, all energies were collected at once, but a nuclide-specific correction for ^123^I was applied.

### Data conversion

**Fig. 1:**
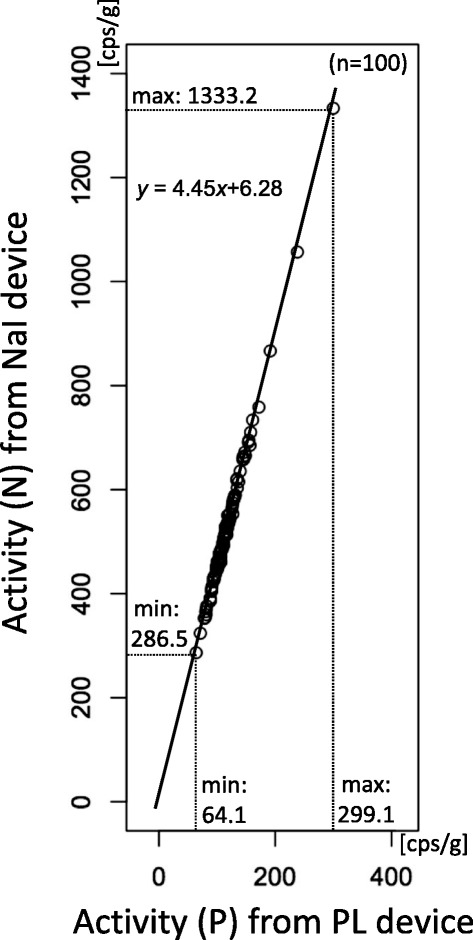
Conversion function from PL (*P*) to NaI (*N*) devices

All of the collected samples were plotted on the graph as shown in Fig. 1. The least-squares (LS) method was applied to obtain the conversion function from the PL counter to the NaI counter. The PL and NaI count pair is designated as (PL_*i*_, NaI_*i*_) where the index for the data point is *i*. The conversion function based on the LS method was defined as:

1 $$ N'=a\;P + b $$

where

2 $$ a = \frac{n{\displaystyle {\sum}_{k=1}^n}{\mathrm{PL}}_k{\mathrm{NaI}}_k-{\displaystyle {\sum}_{k=1}^n}{\mathrm{PL}}_k{\displaystyle {\sum}_{k=1}^n}{\mathrm{NaI}}_k}{{\displaystyle {\sum}_{k=1}^n}{{\mathrm{PL}}_k}^2-{\left({\displaystyle {\sum}_{k=1}^n}{\mathrm{PL}}_k\right)}^2} $$

3 $$ b = \frac{{\displaystyle {\sum}_{k=1}^n}{{\mathrm{PL}}_k}^2{\displaystyle {\sum}_{k=1}^n}{\mathrm{NaI}}_k - {\displaystyle {\sum}_{k=1}^n}{\mathrm{PL}}_k{\mathrm{NaI}}_k\ {\displaystyle {\sum}_{k=1}^n}{\mathrm{PL}}_k}{{\displaystyle {\sum}_{k=1}^n}{{\mathrm{PL}}_k}^2-{\left({\displaystyle {\sum}_{k=1}^n}{\mathrm{PL}}_k\right)}^2} $$

*N*’ is the estimated radioactivity for the NaI device, and *P* is the radioactivity obtained by the PL device.

The resubstitution (R) method was used to determine the conversion function. The leave-one-out (LOO) cross validation method was used to clarify the fluctuation of the error variances because the LOO can separate training cases to configure the conversion function and test cases to test the estimated values based on the conversion function. The statistics software R (ver. 3.11) was used, and the numerical computation was written and was performed in the C language environment.

## Results

The conversion function obtained by using all 100 samples based on the R method was:

4 $$ N' = 4.45\ P + 6.28 $$

**Fig. 2:**
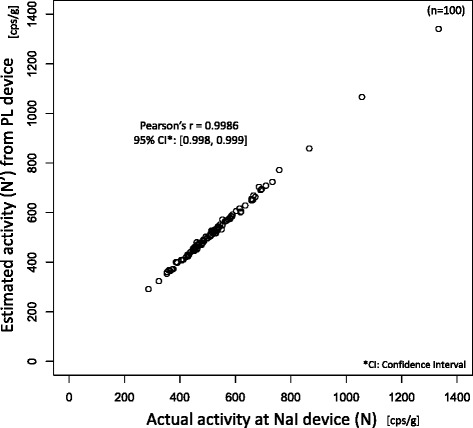
Correlation between actual (*N*) and estimated (*N*’) radioactivities at NaI device

The correlation (Pearson’s *r*) between *N*’ and the radioactivity measurement (*N*) was 0.9986 with the 95 % confidence interval extended from only 0.998 to 0.999 (*p* < 0.001) as shown in Fig. 2. The differences between *N*’ and *N* were tested by using the statistical paired *t* test. The mean and standard deviation (SD) of the difference were 0.174 and 7.326, respectively. The 95 % confidence interval for the mean was from −1.28 to 1.63 including zero (*p* = 0.813).

**Fig. 3:**
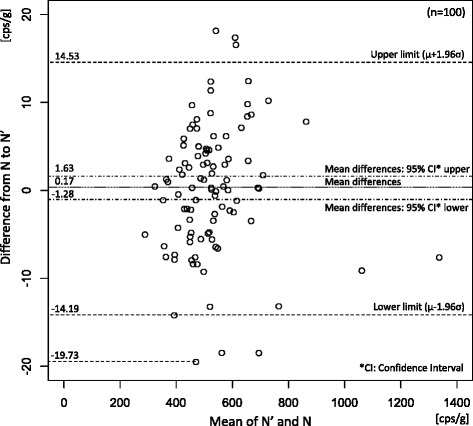
Bland-Altman plot of estimated radioactivities from PL device (*N*’) and actual counts (*N*) by NaI device

Figure 3 shows the Bland-Altman plot between *N*’ and *N*. There is no fixed bias (paired *t* test, *p* = 0.813) and no proportional bias (Correlation test, Pearson’s *r* = 0.032 [−0.165, 0.227], *p* = 0.749).

Based on the R method and the LOO method, the averaged errors/standard deviation/maximum differences/minimum differences between measured radioactivity from the NaI device and the estimated radioactivity *N*’ are shown in Table 1. The errors in the LOO results were slightly larger than those in the R method results. The estimated maximum error ratio of the NaI device to the PL device was 6.9 % (19.73/286.5 [cps/g]). This maximum error ratio was defined as the maximum difference divided by the lowest radioactivity among all the samples from the NaI device.

**Table 1:** Estimated conversion errors in resubstitution (R) and leave-one-out (LOO) methods

Error types	R: resubstitution	LOO: leave-one-out
Average (SD)	0.00 (7.29)	6.32 × 10^−3^ (7.30)
Maximum	17.96	17.98
Minimum	−19.66	−19.73

[cps/g]

## Discussion

Power analysis is the most important discussion for statistical test results [6]. The results of the paired *t* test between *N*’ and *N* showed that the mean and the SD were 0.174 and 7.326, respectively, from 100 samples. Based on these difference parameters, when the probability of alpha error was 0.05, the effect size (*d*) and the power of (1 − *ß*) error probability were derived as 0.0238 and 0.056, respectively, by using G*Power software (Ver. 3.1.9.2) [7, 8].

In general statistics in Cohen’s paper [6], a required sample size *n* (the number of cases) for the paired *t* test is estimated as *n* = 90 when the effect size, the statistical significance, and the power were 0.3, 0.05, and 0.8, respectively, from an a priori analysis. The actual number of sample in this study was 100 to meet the result of this a priori analysis, but the actual effect size, *d* = 0.0238, was much smaller than the general effect size *d* = 0.3.

Based on the actual effect size, the number of cases to show the statistical difference between *N*’ and *N* is estimated as 13,859 with the power of 0.80 from another a priori analysis. This sample size is not a practical value because the average number of examination per year in our university hospital was approximately 100.

**Fig. 4:**
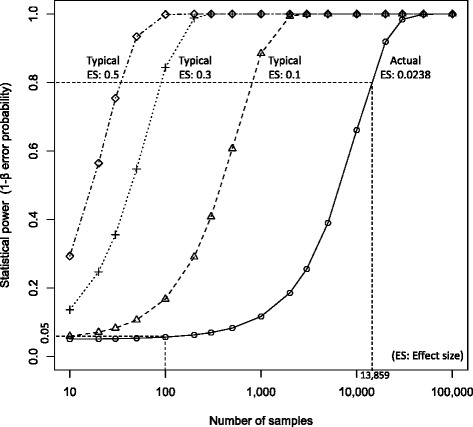
Relationship between statistical power and number of samples at *p* = 0.05 by changing the effect size (ES) from the actual ES = 0.0238 to the typical ES {0.1, 0.3, 0.5}

Figure 4 shows the relationship between statistical power and various effect sizes. Changes of statistical powers with an actual effect size of 0.0238 and three typical effect sizes of 0.1, 0.3, and 0.5 are shown as bold and dashed lines. The statistical power of this study was small (0.05) because of the small effect size of 0.0238. The result suggested that the statistical test might have been prone to the type II error. The effect size depended on the SD of the differences in counts between two measurements. Not only the differences of scintillation materials but also those of counting methods in different devices might have affected the effect size because fluctuation of radioactivity measurements was observed as SD.

We will assume that the power analysis is an important method to clarify the difference between measurements but not to clarify the equivalency between the measuring results from the two counting devices. Our equivalency result will have to depend on (1) the results of the correlation, (2) the system errors obtained by the Bland-Altman plot, and (3) the maximum difference between *N*’ and *N*.

## Conclusion

Between different well counters, a simple conversion function based on the least-squares method can be used for robust data conversion. A hundred blood samples using a fixed type of sample tube and a fixed radionuclide may be required to set up the conversion function and determine the simple error.
